# Spontaneous Retroperitoneal Hematoma Treated with Percutaneous Transarterial Embolization in COVID-19 Era: Diagnostic Findings and Procedural Outcome

**DOI:** 10.3390/tomography8030101

**Published:** 2022-05-01

**Authors:** Francesco Tiralongo, Salvatore Seminatore, Stefano Di Pietro, Giulio Distefano, Federica Galioto, Francesco Vacirca, Francesco Giurazza, Stefano Palmucci, Massimo Venturini, Mariano Scaglione, Antonio Basile

**Affiliations:** 1 Radiology Unit 1, Department of Medical Surgical Sciences and Advanced Technologies “GF Ingrassia”, University Hospital Policlinico “G. Rodolico-San Marco”, 95123 Catania, Italy; uni303093@studium.unict.it (S.S.); s.dipietro@studium.unict.it (S.D.P.); uni314543@studium.unict.it (F.G.); f.va77@libero.it (F.V.); spalmucci@unict.it (S.P.); antonio.basile@unict.it (A.B.); 2 U.O.C. Medicina Interna, Dipartimento di Medicina, Ospedale “R. Guzzardi” di Vittoria, ASP Ragusa, 97100 Ragusa, Italy; giulio.distefano@asp.rg.it; 3 Interventional Radiology Department, Cardarelli Hospital of Naples, 80131 Naples, Italy; francesco.giurazza@aocardarelli.it; 4 Department of Diagnostic and Interventional Radiology, Circolo Hospital, Insubria University, 21100 Varese, Italy; massimo.venturini@uninsubria.it; 5 Department of Radiology, James Cook University Hospital, Middlesbrough TS4 3BW, UK; mariano.scaglione@pinetagrande.it; 6 Teesside University School of Health and Life Sciences, Tees Valley, Middlesbrough TS1 3BX, UK; 7 Department of Radiology, “Pineta Grande” Hospital, Via Domitiana Km. 30, Castel Volturno, 81030 Caserta, Italy; 8 Department of Clinical and Experimental Medicine, University of Sassari, 07100 Sassari, Italy

**Keywords:** COVID-19, SARS-CoV-2, embolization, radiology, interventional, angiography, digital subtraction, hematoma, retroperitoneal space

## Abstract

(1) Background: Spontaneous retroperitoneal hematomas are a relatively common occurrence in hospitalized patients with COVID-19 related pneumonia, and endovascular treatment of trans-arterial embolization (TAE) may be a life-saving procedure after failure of medical and supportive therapy. The aim of our study was to evaluate spontaneous retroperitoneal hematomas in the COVID-19 era, focusing on their imaging features at CTA and DSA and on the safety, as well as technical and clinical success, of TAE, comparing patients affected by COVID-19 and non-COVID-19 patients. (2) Materials and Methods: We retrospectively enrolled 24 patients with spontaneous retroperitoneal hematoma who underwent TAE; of these, 10 were hospitalized for COVID-19-related pneumonia, while the other 14 were without COVID-19 infection. We evaluated the demographic data, hemoglobin values before and after the procedure, preprocedural aPTT, preprocedural INR, diagnostic and interventional imaging findings, procedural outcome (technical success) and survival periprocedural (clinical success), and major and minor complications. (3) Results: The mean age of the study population was 72.7 ± 11.2 years. CTA revealed signs of active bleeding in 20 patients (83%). DSA showed signs of active bleeding in 20 patients (83%). In four patients (17%), blind embolization was performed. The overall technical success rate was 100%. Clinical success was achieved in 17 patients (71%), while seven patients (29%) rebled within 96 h, and all of them were retreated. No major periprocedural complication was reported. The comparison between the two groups did not show statistically significant differences for gender, mean age, mean pre- and postprocedural hemoglobin, aPTT and INR, mean hematoma volume (cm^3^), or mean delay between CT and DSA. Active bleeding at CTA was detected in 90% of COVID-19 patients and 79% of non-COVID-19 patients (*p* = 0.61). At DSA, active bleeding was assessed in eight out of 10 (80%) patients in the COVID-19 group and 12 out of 14 (86%) patients in the non-COVID-19 group (*p* = 1). Technical success was obtained in 100% of patients in both groups. Clinical success rates were 70% for COVID-19 group and 71% for the non-COVID-19 group. We found no statistical significance between the clinical success rates of retroperitoneal spontaneous hematoma embolization in patients with or without SARS-CoV-2 infection. (4) Conclusions: We suggest that, similar to what has been reported in other studies in non-COVID-19 patients, TAE should be considered an important safe, effective, and potentially life-saving option for the management and the treatment of patients affected by COVID-19 who present with spontaneous retroperitoneal hematoma and who could not benefit from conservative treatment.

## 1. Introduction

The first case of the disease known as coronavirus disease 2019 (COVID-19), caused by severe acute respiratory syndrome coronavirus-2 (SARS-CoV-2), was reported by the World Health Organization (WHO) on 31 December 2019. It was first detected in the city of Wuhan, China, and it subsequently rapidly spread around the world, becoming responsible for the coronavirus disease pandemic and causing more than 5.9 million deaths [1,2,3].

The clinical spectrum of COVID-19 varies from asymptomatic or paucisymptomatic forms to clinical illness characterized by acute respiratory failure requiring mechanical ventilation, septic shock, and multiple organ failure. Most symptomatic patients, however, commonly present with fever, dry cough, and malaise, but other symptoms such as diarrhea, hemoptysis, and headache are not uncommon [4].

SARS-CoV-2 primarily affects the respiratory tract, but pathophysiology includes endothelial dysfunction, as well as a hypercoagulable state and, therefore, a high incidence of thromboembolic complications [5,6,7]. Although thrombosis and hypercoagulability are hallmarks of this disease, the presence of thrombocytopenia, a hyper-fibrinolytic state, consumption of coagulation factors, and cytokine storm increases the risk of developing spontaneous bleeding in COVID-19 patients [6,8].

The thromboprophylactic administration of anticoagulants with low-molecular-weight heparin (LMWH) to prevent thrombotic events and organ damage can result in bleeding manifestations such as hematomas or active bleeding [6].

Spontaneous retroperitoneal hematoma (SRH) is defined as bleeding into the retroperitoneal space without associated trauma or iatrogenic manipulation [9], and the iliopsoas muscle, which refers to the joined psoas and the iliacus muscles, is the most common site for this bleeding [10].

There is a lack of focused studies about the incidence or prevalence of this condition, but it has been reported that hematomas of the psoas muscle occur in 0.1–0.6% of patients who have risk factors such as elderly age and undergoing anticoagulation therapy or hemodialysis [11].

In fact, SRH is considered a rare complication in patients undergoing anticoagulant therapy and a potentially life-threatening condition. Spontaneous retroperitoneal bleeding may also occur in patients with coagulation disorders, malignancies, hematologic diseases, and trauma [12].

It has been assessed that SRH is a possible complication occurring in COVID-19 patients on anticoagulant therapy; in fact, the incidence of spontaneous muscle hematoma is 3–4 times higher in COVID-19 patients undergoing anticoagulant therapy than in patients on anticoagulant therapy without COVID-19 [13].

The diagnosis of SRH can be difficult since signs and symptoms are often vague and nonspecific. Abdominal pain and pain the back, flank, and hip are the most common symptoms. Clinical and laboratory signs of bleeding and hypovolemia could also be present, especially in patients with massive bleeding, but physical examination is generally nondiagnostic, and imaging tests are essential to achieve the diagnosis [14].

Computed tomography angiography (CTA) is the gold standard for the identification of SRH, allowing a rapid diagnosis of retroperitoneal hematoma and localization of the site of the bleeding, as well as the extension of the hematoma and the exclusion of other acute abdominal diseases. CTA, moreover, has a key role in the evaluation of the presence of active bleeding within the hematoma, being able to detect bleeding with a flow rate as low as 0.3 mL/min, and to guide the subsequent management of this condition and the therapeutic iter, since the detection of ongoing bleeding within the hematoma has been associated with unsuccessful conservative treatment [11,15,16,17].

**Figure 1:**
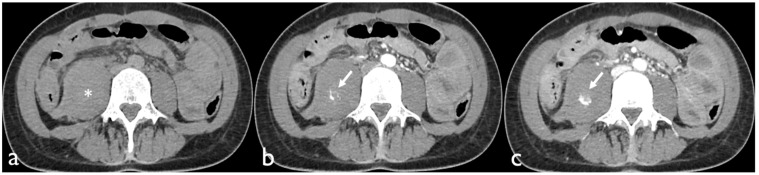
Non-contrast CT image (**a**) of a patient presenting with a hematoma of the right iliopsoas muscle extending into the retroperitoneal space (*). Contrast-enhanced axial CT images show active bleeding in the arterial (**b**) and portal phase (**c**).

Active bleeding on CTA can be recognized as a focal area of higher density (with similar HU values to the abdominal aorta or major adjacent arteries) within the hematoma in arterial phase images, which is enlarged in venous phase images and not present in baseline unenhanced images (Figure 1) [18].

On an unenhanced CT scan, a recent hematoma appears as a soft tissue density mass (30–50 HU), which sometimes dislocates the adjacent structures and, after some time, could present the “hematocrit effect” sign, which is a fluid–fluid level in the context of the hematoma generated by the stratification of cellular elements heavier than serous fluid supernatant [15].

**Figure 2:**
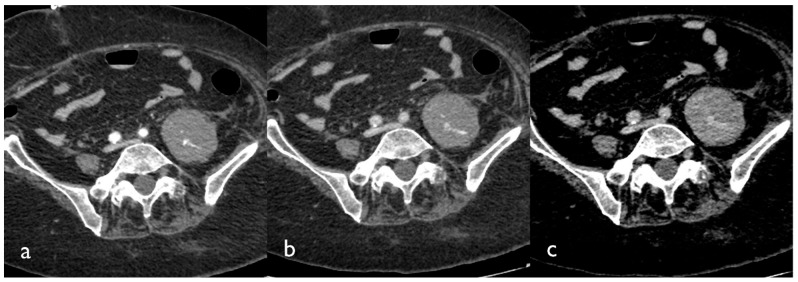
CT images of a patient presenting with a large hematoma of the left iliopsoas muscle. Tri-phase contrast-enhanced axial CT images show contrast blush in the arterial (**a**), portal (**b**), and delayed phase (**c**); active bleeding is seen like small hyperdense foci within the hematoma that appear in the arterial phase and grow in the venous and delayed phase.

If active bleeding occurs within a hematoma with fluid–fluid level, it could be possible to identify the “signal flare” sign, which is generated by the layering of extravasated contrast medium between cellular and fluid components of the hematoma, due to the different gravitational weight (Figure 2) [15].

The management and treatment of SRH, however, are still controversial since there are no specific guidelines to suggest the best option. In patients hemodynamically stable with no evidence of active bleeding, conservative management is usually recommended. This includes volume resuscitation, withdrawal or reversal of anticoagulation therapy, vasoactive drugs, and transfusions. In hemodynamically unstable patients or in the case of failure of conservative treatment, endovascular treatments such as digital subtraction angiography (DSA) and subsequent percutaneous transarterial embolization (PTAE) of the bleeding vessels have been recognized as a valid, effective, and potentially life-saving option in these patients, while open surgery is generally considered in very selective cases since the surgical removal of the hematoma may disturb the tamponade effects of the retroperitoneum, resulting in increased bleeding [19,20,21].

The aim of our study was to evaluate spontaneous retroperitoneal hematomas in the COVID-19 era, focusing on their imaging features at CTA and DSA and on the safety, as well as technical and clinical success, of percutaneous transarterial embolization in the treatment of this condition, comparing patients affected by COVID-19 and non-COVID-19 patients.

## 2. Materials and Methods

### 2.1. Study Population and Setting

We retrospectively analyzed the files from our RIS (Radiology Information System) and PACS (Picture Archiving and Communication System) system of all consecutive patients with or without confirmed COVID-19 infection who presented with spontaneous retroperitoneal hematoma and who underwent emergency angiographic evaluation and embolization between March 2020 and January 2022.

Overall, the study population included 24 patients. Furthermore, we divided all patients into two groups depending on the presence or absence of confirmed SARS-CoV-2 infection (COVID-19 group vs. non-COVID-19 group) to analyze the differences between the two study populations and to compare clinical and technical success rates and the rates of complications between these groups and these approaches.

Clinical and laboratory data such as pre- and postprocedural hemoglobin, preprocedural activated partial thromboplastin time (aPTT), and preprocedural international normalized ratio (INR) were obtained from the digital medical records.

Low-molecular-weight heparin (LMWH) was administered to all patients with SARS-CoV-2 infection for either prophylactic or therapeutic reasons, according to local protocol.

### 2.2. Pre-Interventional CTA

All patients underwent CTA before DSA. The CT examinations were performed using two different scanners: Optima 660 (GE Healthcare, Chicago, IL, USA) and Toshiba Aquilon Prime (Toshiba Medical Systems Corporation, Otawara, Japan).

The CT protocol consisted of a non-contrast phase followed by an arterial phase, acquired with an automatic bolus tracking technique with the ROI (region of interest) placed in the proximal abdominal aorta, a venous phase (acquired with a delay of 60 s), and a delayed phase (acquired with a delay of 120 s).

A bolus of 80–120 mL of Iomeprol (Iomeron 350 or 400 mgI/mL; Bracco Imaging, Milan, Italy) followed by 30 mL of saline flush was administrated using an automated contrast injection system at a flow rate of 4–5 mL/s. The CT images were reformatted in both coronal and sagittal planes.

The location of the hematoma and the extension of the hematoma into the iliopsoas muscle or into the retroperitoneal space were assessed. For each patient, the volume (cm^3^) of the retroperitoneal hematoma was assessed on the basis of CTA images using the Livewire Mode Segmentation Tool integrated in our PACS (Vue PACS version 12.1; Carestream Health, Rochester, NY, USA), defining the contour in a semi-automatic manner in order to volumetrically measure the hematoma.

Signs of active bleeding (active extravasation of contrast medium) or vascular injuries (i.e., pseudoaneurysm formation) in the hematoma were evaluated on CTA; active bleeding was defined as the presence of contrast material extravasation that appeared on arterial-phase CT images and grew on venous- and late-phase images.

In patients without signs of active bleeding at CTA, it was decided to proceed with DSA evaluation and eventual embolization because of unstable hemoglobin levels or because of the continuous need for transfusion to maintain acceptable hemoglobin levels (>8.0 g/dL).

### 2.3. Diagnostic Angiography and Transarterial Embolization

DSA and TAE were performed by interventional radiologists with at least 5 years of experience and by radiology residents in their third or fourth year of training. DSA and endovascular treatments were performed using a Toshiba Infinix-i CAS-880A Cath Angio Lab (Toshiba Medical Systems Corporation, Otawara, Japan).

In COVID-19 group, about 20 min before the expected time of patient arrival, the interventional team set in motion all the prevention measures to avoid contagion; all the materials necessary for the procedure, planned on the basis of the CT images, were placed in the room on a specific table, and all the cabinets containing the material were sealed in order to avoid contamination. Each operator involved in the procedure began the dressing maneuvers in a separate room from the angiography room, defined as “clean”. The dressing procedure took place in a very specific order in order to subsequently facilitate undressing and avoid contamination.

For both groups, after skin disinfection and sterile draping, a 10 cm long 5 Fr guiding sheath was introduced via the right (preferred) or left common femoral artery. Arteriography was performed with Iomeprol (Iomeron 350, Bracco Imaging, Milan, Italy) as the contrast agent with a frame rate between two and six images per second.

For patients in whom pre-interventional CTA did not show active bleeding or the target vessel was not clearly identified, overview angiograms with a 4 or 5 Fr catheter placed in the abdominal aorta were performed. In all other cases, CTA-guided selective arteriography of the most likely bleeding site was immediately performed. Selective catheterization was performed with the use of 4 or 5 Fr appropriate diagnostic catheters, such as Cobra 2 (Cordis, Santa Clara, CA, USA) or Sos-Omni (AngioDynamics, Latham, NY, USA) and an angled, flexible 0.035 inch hydrophilic guidewire (Radiofocus; Terumo, Tokyo, Japan). Super selective or distal catheterization was usually conducted with a 2.7 Fr preloaded microcatheter system, which was inserted coaxially into the diagnostic catheter.

Findings considered for angiographic proof of bleeding were direct signs of active bleeding (contrast blush extravasation, focal spot of enhancement, hemorrhagic petechiae), indirect signs (vessel cut-off sign), pseudoaneurysm formation, and absence of active bleeding.

Embolization was carried out via the microcatheter using pushable or detachable coils (deployed coil diameter: 2–6 mm), a temporary embolic agent such as re-absorbable gelatin sponge (Spongostan^®^, Johnson & Johnson Medical N.V., New Brunswick, NJ, USA), or a combination of coils and gelatin sponge. The choice between the embolic materials was determined by the discretion of the treating interventional radiologist and considering the vascular anatomy and the position of the microcatheter within the target vessel. The microcatheter was placed as close as possible to the bleeding site to release the embolic material, and, after embolization, the adjacent vessels were catheterized selectively to exclude the presence of collateral vascular flow to the bleeding source. At the end of the procedure, the guiding sheath was left in place in case of recurrent bleeding for 24–48 h. The patient was transported to the intensive care unit (ICU) and, for the COVID-19 group, the interventional radiology team started the undressing maneuver, and the angiographic room was sanitized.

Target embolized vessels, embolic materials, the number of embolic materials used in the TAE, the procedural timing (more or less than 60 min), and the time between preprocedural CTA and angiography were assessed.

### 2.4. Laboratory Findings

The following laboratory values were recorded: pre- and postprocedural hemoglobin, preprocedural activated partial thromboplastin time (aPTT), and preprocedural international normalized ratio (INR). The pre-interventional values were gathered at a maximum of 12 h before the embolization procedure. The corresponding post-interventional values were gathered within 24 h after the procedure.

### 2.5. Definitions

A confirmed case of COVID-19 was defined by a positive real-time reverse-transcription PCR (RT-PCR) assay for SARS-CoV-2 on a nasopharyngeal swab.

Targeted embolization was defined as the embolization of vessels where direct or indirect signs of active bleeding were demonstrated on DSA study.

Blind embolization was defined as embolization of a target vessel without angiographic proof of extravasation, typically guided by CTA findings in normal-appearing vessels [22].

Technical success was defined as complete embolization of all target vessels.

Clinical success was defined as the absence of clinical, laboratory, or radiological signs of rebleeding within a 96 h window after the procedure. The 96 h follow-up was based on clinical and laboratory parameters and, when indicated, CTA evaluation. Clinical failure was considered in patients who presented signs of rebleeding (hemoglobin decrease, hypovolemic shock, or evidence of persistent bleeding at postprocedural CTA examination) and who needed to undergo a new angiographic procedure followed by embolization of bleeding vessels. Minor and major procedure-related complications were defined according to the Society of Interventional Radiology [23].

### 2.6. Statistics

Statistical analysis was performed using the MedCalc program (MedCalc version 11.4.4.0, MedCalc Software bvba, Mariakerke, Belgium). Continuous variables are presented as the mean ± SD. Categorical variables are reported as percentages. Characteristics of the study population are reported as the mean (SD), range, and median.

For comparative statistics between the COVID-19 group and non-COVID-19 group, gender percentage, active bleeding at CTA and DSA rate, muscular fascia rupture percentage, procedural timing, clinical success rates, and rates of complications, the Fisher exact test was performed. The comparison between mean values of age, pre- and postprocedural hemoglobin, aPTT, and INR, hematoma volume, mean embolized arteries per patient, mean embolic materials used, and delay between CT and DSA was assessed using an independent-sample *t*-test. A *p*-value <0.05 was considered statistically significant.

## 3. Results

### 3.1. Study Population and Laboratory Findings

A total of 24 patients (71% male, mean age of 72.7 ± 11.2 years) with spontaneous retroperitoneal hematoma were enrolled in the study. The mean value of preprocedural hemoglobin in our population was 7.96 ± 1.35 g/dL. The mean preprocedural aPTT was 38.95 ± 15.68. The mean preprocedural INR was 1.30 ± 0.27. The mean postprocedural hemoglobin was 8.75 ± 1.05 g/dL. The characteristics of the study population are summarized in Table 1.

### 3.2. Pre-Interventional CTA

CT angiography showed that the hematoma was localized within the iliopsoas muscle in all (100%) patients. Mean hematoma volume was 865.7 ± 440.67 cm^3^ (range 228–1690.7 cm^3^).

Extension into the retroperitoneal space, outside the iliopsoas muscle, was present in 19 (79%) cases. CTA revealed signs of active bleeding within the hematoma in 20 patients (83%). In four patients (17%), CTA did not show signs of active bleeding (Table 1).

### 3.3. Diagnostic Angiography and PTAE

Angiography and TAE were performed with a mean delay of 8.82 ± 5.98 h (range 1–22.5 h) from CT.

DSA showed direct signs of active bleeding in 20 patients (83%). In four patients (17%), no angiographic evidence of active bleeding was found (Table 1); three of these patients showed active bleeding at CTA, while one showed no signs of active bleeding at CTA.

The embolized arteries were the lumbar artery (*n* = 19), iliolumbar artery (*n* = 17), deep circumflex iliac artery (*n* = 5), intercostal artery (*n* = 2), and epigastric inferior artery (*n* = 1).

A total of 69 arteries were embolized, with a mean value of 2.87 ± 1.75 arteries per patient.

Embolization materials used were absorbable gelatin foam (Spongostan^®^; Johnson and Johnson, New Brunswick, NJ, USA) in 11 (46%) patients and a combination of gel foam and coils in 13 (54%) patients.

Blind embolization was performed in four patients (17%). The mean time for the angiographic procedure was less than 60 min in 37.5% of the procedures (nine out of 24), exceeding 60 min in 62.5% (15 out of 24).

The technical success rate was 100%. Clinical success was achieved in 17 patients (71%) with no need for further interventions. Seven patients (29%) rebled during follow-up (96 h time window), and all of them were retreated with a new TAE procedure: four patients had a recurrence within 24 h of DSA, two patients rebled after 48 h, and one patient had a longer gap before rebleeding at 96 h.

No major periprocedural complication was reported. One patient (4%) had a minor complication: dissection flap of deep circumflex iliac artery during the selective catheterization that did not need any specific treatment (Table 2).

### 3.4. COVID-19 vs. Non-COVID-19 Groups

The characteristics of the study populations of the two groups are summarized in Table 1. The comparison between the two groups did not show statistically significant differences for gender, mean age, mean pre- and postprocedural hemoglobin, aPTT, and INR, mean hematoma volume (cm^3^), and mean delay between CT and DSA.

Signs of active bleeding at CTA were detected in 90% of COVID-19 patients and 79% of non-COVID-19 patients, with no statistically significance difference (*p* = 0.61).

Moreover, at DSA, active bleeding was assessed in eight out of 10 patients (80%) in the COVID-19 group and 12 out of 14 (86%) patients in the non-COVID-19 group, with no statistically significant difference (*p* = 1).

The independent-sample *t*-test, for comparing the mean embolized arteries and the mean number of embolic materials (coils) used in the TAE, showed no statistical difference between the two groups (Table 3).

Likewise, the procedural time was <60 min in 30% of procedures in the COVID-19 group and in 43% of procedures in the non-COVID-19 group, without statistical significance according to the Fisher exact test (*p* = 0.67).

**Figure 3:**
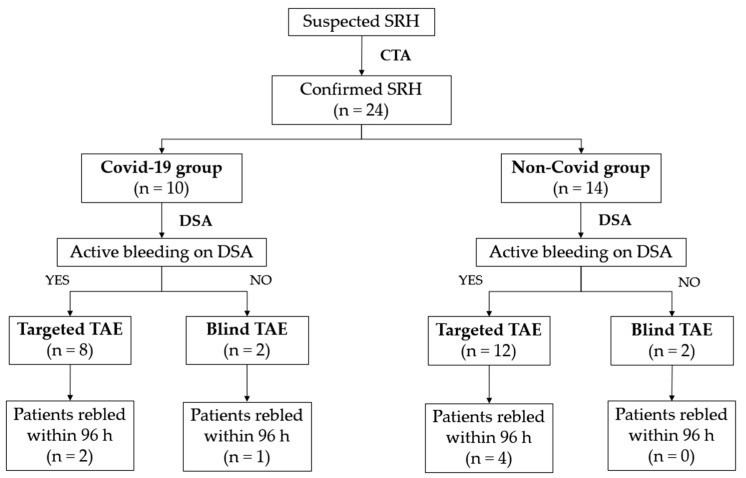
Patient flowchart.

Technical success was obtained in 100% of patients in both groups. Clinical success rates were 70% for the COVID-19 group and 71% for the non-COVID-19 group; three out of 10 (30%) SARS-CoV-2-infected inpatients and four out of 14 (29%) patients without SARS-CoV-2 infection rebled after the first TAE (Figure 3). The Fisher exact test showed no statistical significance between the clinical success rates of retroperitoneal spontaneous hematoma embolization in patient with or without SARS-CoV-2 infection (Table 3).

## 4. Discussion

Spontaneous retroperitoneal bleeding is a rare but potentially fatal complication of patients under anticoagulant therapy and should be considered in those patients that present painful mass and hypotension. The mortality rate in the current literature is about 26.8% [24], with an incidence ranging from 0.1% to 0.6% [25].

The COVID-19-specific incidence of spontaneous muscular hematoma is actually unclear; Riu et al. reported an incidence of 1.95% of soft-tissue spontaneous bleeding [20], higher than observed in general hospitalized patients receiving low-molecular-weight heparin (LMWH) for some reason. An explanation could be found in the physiopathology of SARS-CoV-2 infection related to endothelial damage [26,27].

SARS-CoV-2 infection carries a special condition of endotheliitis both for the viral infection of the cell and for the inflammatory response with subsequent cytokine storm, with the consequence of micro and macro thrombosis, as well as endothelial injury leading to micro-vessel fragility [20]. Endothelial damage was the reason why hospitalized patients were advised to treat hospitalized patients with high doses of LMWH (especially in the first wave, preventive doses are currently preferred), and why it is also difficult to achieve hemostasis in case of bleeding. Moreover, the mobilization of the patient in the ICU, the coughing, the vomiting, and the breathing exercises with pronation, part of the therapy of COVID-19, as a muscle isometric effort, can also lead to microtrauma and bleeding [28].

As mentioned before, despite the lack of specific guidelines on the treatment of spontaneous retroperitoneal hematoma, recent studies suggest that TAE plays an important role in the management of this condition, representing a safe and effective option for the treatment of spontaneous abdominal wall hematomas, including retroperitoneal hematomas [11,21]. Hemodynamically unstable patients can be also treated with TAE, due to its minimally invasive and quick therapeutic effect, compared to surgical treatment [20].

In the COVID-19 setting, PTAE can be considered an important tool in the treatment of spontaneous bleeding, including retroperitoneal hematoma, occurring in COVID-19-infected inpatients under anticoagulant therapy [20]. Lucatelli et al. recently suggested the use of PTAE in COVID-19 patients, preferring it to conservative treatment and encouraging selective embolization even without signs of active bleeding [29].

In our study, spontaneous retroperitoneal bleeding occurred in elderly patients (mean age 72.7 ± 11.2 years), consistent with some studies found in the literature, in which a mean age ranging between 70 and 71.9 years was reported [21,30]. In our COVID-19 group, the mean age was 75 ± 10.5 years, not significantly greater than our population of non-COVID patients (*p* = 0.71), but the difference could be explained by the generally higher mean age of patients admitted to the hospital because of COVID-19 infection, according to the data presented by the Italian National Institute of Health [31].

Multiphasic CT represents the gold standard for the identification of a spontaneous retroperitoneal hematomas [15]. CTA assessed in the preprocedural setting allows recognizing CT signs of active bleeding and identifying the bleeding vessel, thus providing valuable assistance to the interventional radiologist in planning the embolization procedure, and influencing the timing of therapeutic treatment [11,15].

In our experience, CTA revealed signs of active bleeding in 20 patients (83%), specifically, 90% (9/10) of COVID-19 patients and 79% (11/14) of non-COVID-19 patients, with no statistically significant difference between the two groups. Our results are in line with the rates reported in the literature, ranging from 78.9–93.3% [21,24,32].

Furthermore, CTA represents an essential tool for the evaluation of the extent of the hematoma and its volume. In our study, the mean hematoma volume was 865.7 ± 440.67 cm^3^, without a significant difference between the COVID-19 (953.75 cm^3^) and non-COVID-19 (802.82 cm^3^) groups (*p* = 0.42). These results are in agreement with the findings of Barral et al., who reported a mean hematoma volume of 862 ± 618 cm^3^ [24]. As described by Riu et al., the volume of hematoma detected at CT was the most significant factor for choosing PTAE [20], and a hematoma volume >1022 cc was considered a predictive factor of short-term outcome [24].

The extension of the hematoma into the retroperitoneal space, outside the iliopsoas muscle, was reported in 79% of patients. This finding is associated with a high mortality rate [24], might predict continued bleeding even after discontinuing anticoagulation therapy, and is generally correlated to massive bleeding even after the treatment [33].

On DSA, active bleeding was detected in 83% (20/24) of patients (80% in COVID-19 group and 86% in non-COVID-19 group, *p* = 1), much higher than the findings of Klausenitz et al. and Tani et al., who detected active bleeding in DSA in 73.3% and 78.9% of patients, respectively [21,32].

In our series, the vessels most frequently embolized were the lumbar and iliolumbar arteries (19 and 17 times, respectively), in line with the observations of Klausenitz et al. and Tani et al. [21,32].

The average number of arteries embolized was 2.87 ± 1.75, and the comparison between the two groups showed no significant differences for the mean of embolized arteries, indicating that, in COVID-19 patients, the embolization approach was similar to that in non-COVID-19 patients.

The high number of embolized vessels per patient underlies the complex vascularization of retroperitoneal hematomas, with multiple anastomoses between lumbar and iliolumbar arteries; this condition requires embolization of the target vessels associated with embolization of arteries of the same territory, i.e., the over- and underlying lumbar arteries and iliolumbar artery [24].

As reported in another study [34], the most common embolic material used in our study was a combination of coils and gel foam, and this finding was maintained across both groups; when DSA showed no signs of active bleeding, empirical or “blind” embolization was always performed. It is worth noting that a recent study published by Di Pietro et al. suggested that blind embolization is comparable to targeted (or non-bind) embolization approach, in terms of efficacy and safety, in the treatment of spontaneous abdominal wall hematomas, including retroperitoneal hematomas [11].

Furthermore, the blind embolization approach is especially encouraged in COVID-19 patients, as reported by Lucatelli et al., who also suggested the use of an adjunctive embolic agent (gel foam/glue) to facilitate clot formation, as well as the use of selective embolization even without signs of active bleeding [29].

Moreover, the COVID-19 group showed no differences, compared to the non-COVID-19 group, in terms of mean time between CTA and DSA and mean duration of the procedure.

Our study reported a technical success rate of 100% and a clinical success rate of 71%. Therefore, seven patients had evidence of rebleeding within a 96 h interval, and they were all retreated with a new TAE procedure. The clinical success rate of the secondary TAE treatment was 100%. Our results are consistent with those found in other published studies, where technical success rates ranged from 96% to 100% and clinical success rates ranged from 65% to 93% [21,24,34].

The comparison of technical and clinical success rates between the COVID-19 group and non-COVID-19 group showed no statistically significant differences among them. The technical success rates were 100% in both groups, and the clinical success rates were comparable (70% and 71%, respectively).

We recognize that this study had several limitations. Mainly, this was a retrospective single-center study with a limited number of included patients (especially COVID-19 patients); secondly, we could not assess if non-COVID-19 patients were administered anticoagulant or antiplatelet drugs, since these data were not available on our digital medical records or on our RIS/PACS system. A further limitation of the study (secondary to the retrospective design) was the lack of long-term mortality assessment, since patients were followed up for only 96 h after the procedure for clinical purposes; however, these limitations had a limited impact on the scope of the work as the endovascular treatment of supplied hematomas is to be considered an emergency procedure in conditions of quoad vitam risk.

## 5. Conclusions

The results obtained from our study revealed no significant differences in terms of technical and clinical success rates and complications for TAE performed to treat spontaneous retroperitoneal hematomas in patients affected by COVID-19 and in patients without COVID-19.

Moreover, our study confirmed that TAE is a safe and effective option for the treatment of SRH, highlighting the importance of the preprocedural CTA to localize the site of the bleeding, the extension of the hematoma, and the presence of signs of active bleeding, to exclude other acute abdominal diseases and to plan the DSA and the subsequent TAE procedure.

Furthermore, since, as it has been suggested by some authors [29] that SRH presenting in COVID-19 patients differs from that presenting in non-COVID-19 patients because of conservative management often failing in the former, the findings of this work could help to better define the approach in the treatment of COVID-19 patients affected by SRH. Our study, in fact, confirms that TAE should be considered an important, safe, effective, and potentially life-saving option for the management and the treatment of patients affected by COVID-19 who present with spontaneous retroperitoneal hematoma and who could not benefit from conservative treatment.

However, further studies are necessary to prospectively compare embolization treatment to conservative management and open surgery in spontaneous retroperitoneal hematomas and to deepen the knowledge about the role of the blind embolization approach in those cases where no active bleeding can be identified on DSA.

## Figures and Tables

**Table 1:** Characteristics of our study population and findings at CTA and DSA.

Characteristic		Value
**Age**	Mean ± SD	72.7 ± 11.2 years
**Male**		17 (71%)
**Female**		7 (29%)
**Preprocedural hemoglobin (g/dL)**	Mean ± SD	7.96 ± 1.35
**Preprocedural aPTT**	Mean ± SD	38.95 ± 15.68
**Preprocedural INR**	Mean ± SD	1.30 ± 0.27
**Postprocedural hemoglobin (g/dL)**	Mean ± SD	8.75 ± 1.05
**CT findings**		
Active bleeding		20 (83%)
No proof of active bleeding		4 (17%)
**Mean hematoma volume (cm^3^)**	Mean ± SD	865.7 ± 440.67 cm^3^
**Extension into the retroperitoneal space**		19 (79%)
**Angiographic findings**		
Direct signs of active bleeding		20 (83%)
Indirect signs of bleeding		0 (0%)
No proof of active bleeding		4 (17%)
Mean embolized arteries		2.87 ± 1.75

**Table 2:** Outcome of PTAE, embolized arteries, and timing of rebleeding after TAE.

Outcome of percutaneous transarterial embolization		Value *n* (%)
	Technical success rate	24 (100%)
	Clinical success rate	17 (71%)
	Major Complications	0 (0%)
	Minor Complications	1 (4%)
Embolized arteries		Value *n* (%)
	Lumbar artery	19 (43%)
	Iliolumbar artery	17 (39%)
	Deep circumflex iliac artery	5 (11%)
	Intercostal artery	2 (5%)
	Epigastric inferior artery	1 (2%)
Timing of rebleeding after TAE		Value *n* (%)
	Within 24 h	4 (57%)
	Between 24 to 48 h	2 (29%)
	Between 48 to 72 h	0 (0%)
	Between 72 to 96 h	1 (14%)

**Table 3:** Comparative results between COVID-19 and non-COVID-19 groups.

	COVID-19 Group	Non-COVID-19 Group	*p*-Value
**Number of patients**	10	14	*-*
Male	8 (80%)	9 (64%)	*0.65*
Female	2 (20%)	5 (36%)	*0.65*
**Mean age of patients (years)**	75 ± 10.5	73.2 ± 12.6	*0.72*
**Mean preprocedural hemoglobin (g/dL)**	7.64 ± 1.27	8.2 ± 1.41	*0.33*
**Mean preprocedural INR**	1.35 ± 0.24	1.26 ± 0.3	*0.42*
**Mean preprocedural aPTT**	33.88 ± 6.8	42.46 ± 19.16	*0.21*
**Mean postprocedural hemoglobin (g/dL)**	8.71 ± 1.07	8.78 ± 1.07	*0.86*
**CTA findings**			
Active bleeding at CTA	9 (90%)	11 (79%)	*0.61*
Hematoma volume (cm^3^)	953.75 ± 413.61	802.82 ± 463.57	*0.42*
Extension into the retroperitoneal space	7 (70%)	12 (86%)	*0.61*
**Mean CT-DSA delay (h)**	6.75 ± 2.5	10.30 ± 7.3	*0.15*
**DSA findings**			
Active bleeding at DSA	8 (80%)	12 (86%)	*1*
Mean number of embolized arteries	2.4 ± 1.26	3.21 ± 2	*0.27*
Mean number of embolic materials	2.57 ± 1.5	1.57 ± 0.5	*0.12*
Procedural timing (<60 min)	3 (30%)	6 (43%)	*0.67*
**Technical success rate**	10 (100%)	14 (100%)	*-*
**Clinical success rate**	7 (70%)	10 (71%)	*1*
**Major complications rate**	0 (0%)	0 (0%)	*0.41*

## Data Availability

Not applicable.
